# Chromosomal mosaicism detected by karyotyping and chromosomal microarray analysis in prenatal diagnosis

**DOI:** 10.1111/jcmm.16080

**Published:** 2020-11-17

**Authors:** Yi Zhang, Mei Zhong, Dezhong Zheng

**Affiliations:** ^1^ Department of Obstetrics and Gynecology Nanfang Hospital Southern Medical University Guangzhou China; ^2^ Department of Cardiology Third Affiliated Hospital of Southern Medical University Southern Medical University Guangzhou China

**Keywords:** chromosomal microarray analysis, chromosomal mosaicism, genetic counselling, karyotyping, prenatal diagnosis

## Abstract

To investigate the incidence and clinical significance of chromosomal mosaicism (CM) in prenatal diagnosis by G‐banding karyotyping and chromosomal microarray analysis (CMA). This is a single‐centre retrospective study of invasive prenatal diagnosis for CM. From 5758 karyotyping results and 6066 CMA results, 104 foetal cases with CM were selected and analysed further. In total, 50% (52/104) of foetal cases with CM were affected by ultrasound‐detectable phenotypes. Regardless of whether they were singleton or twin pregnancies, isolated structural defects in one system (51.35%, 19/37 in singletons; 86.67%, 13/15 in twins) and a single soft marker (18.92%, 7/37 in singletons; 13.33%, 2/15 in twins) were the most common ultrasound anomalies. Mosaic autosomal trisomy (19.23%, 20/104) was the most frequent type, and its rate was higher in phenotypic foetuses (28.85%, 15/52) than in non‐phenotypic foetuses (9.62%, 5/52). There was no difference in mosaic fractions between phenotypic and non‐phenotypic foetuses based on specimen sources or overall classification. Discordant mosaic results were observed in 16 cases (15.38%, 16/104) from different specimens or different testing methods. Genetic counselling and clinical management regarding CM in prenatal diagnosis remain challenging due to the variable phenotypes and unclear significance. Greater caution should be used in prenatal counselling, and more comprehensive assays involving serial ultrasound examinations, different specimens or testing methods verifications and follow‐up should be applied.

## 1 INTRODUCTION

Chromosomal mosaicism (CM) is a biological phenomenon defined as an individual who has arisen from a single zygote and has two or more populations of cells with distinct genotypes.^1^ The main underlying mechanisms leading to mosaicism formation involve mitotic or meiotic non‐disjunction errors, anaphase lagging and trisomy rescue, endoreplication events, and uniparental disomy (UPD) associated with trisomy rescue.^2^ Theoretically, the pattern of the mosaic distribution in the foetus and placenta is largely determined by the time, stage and location of these mechanisms occurring during embryonic development.^3^ In clinical practice, the phenotypic effects of CM are generally considered to be highly associated with this mosaic distribution pattern.

Due to the variable and unpredictable distribution patterns of abnormal cell lineages, CM is a challenging issue in prenatal diagnosis. In particular, low‐level mosaicism (<15%‐20%), foetal‐placental discrepancies and the UPD generated by embryo rescue have been reported to be responsible for the increased risk of erroneous diagnoses.^4^, ^5^ Recently, improvements have been made in the field of molecular genetics technologies such as quantitative fluorescent polymerase chain reaction (QF‐PCR), fluorescence in situ hybridization analysis (FISH), CMA and next‐generation sequencing so that supplementary analyses on amniotic fluid or cord blood are often performed to confirm the true foetal involvement and its clinical significance.^6^, ^7^, ^8^, ^9^, ^10^ Nevertheless, even CM thought to be confined to the placenta may reflect a cryptic foetal mosaicism that may or may not give rise to phenotypic consequences, or lead to placental dysfunction related to foetal growth restriction.^11^, ^12^

Thus, based on the unclear clinical significance of CM and limited data about CM in prenatal diagnosis, it is necessary to understand more comprehensively the potential correlation between CM and its phenotypic effects. In this study, we explored the incidence and characteristics of CM detected by G‐banding karyotyping and/or CMA in 104 foetal cases, analysing their phenotypic features, and further comparing the discordant CM results identified from different specimens or different testing technologies.

## 2 MATERIALS AND METHODS

### 2.1 Case selection

**FIGURE 1:**
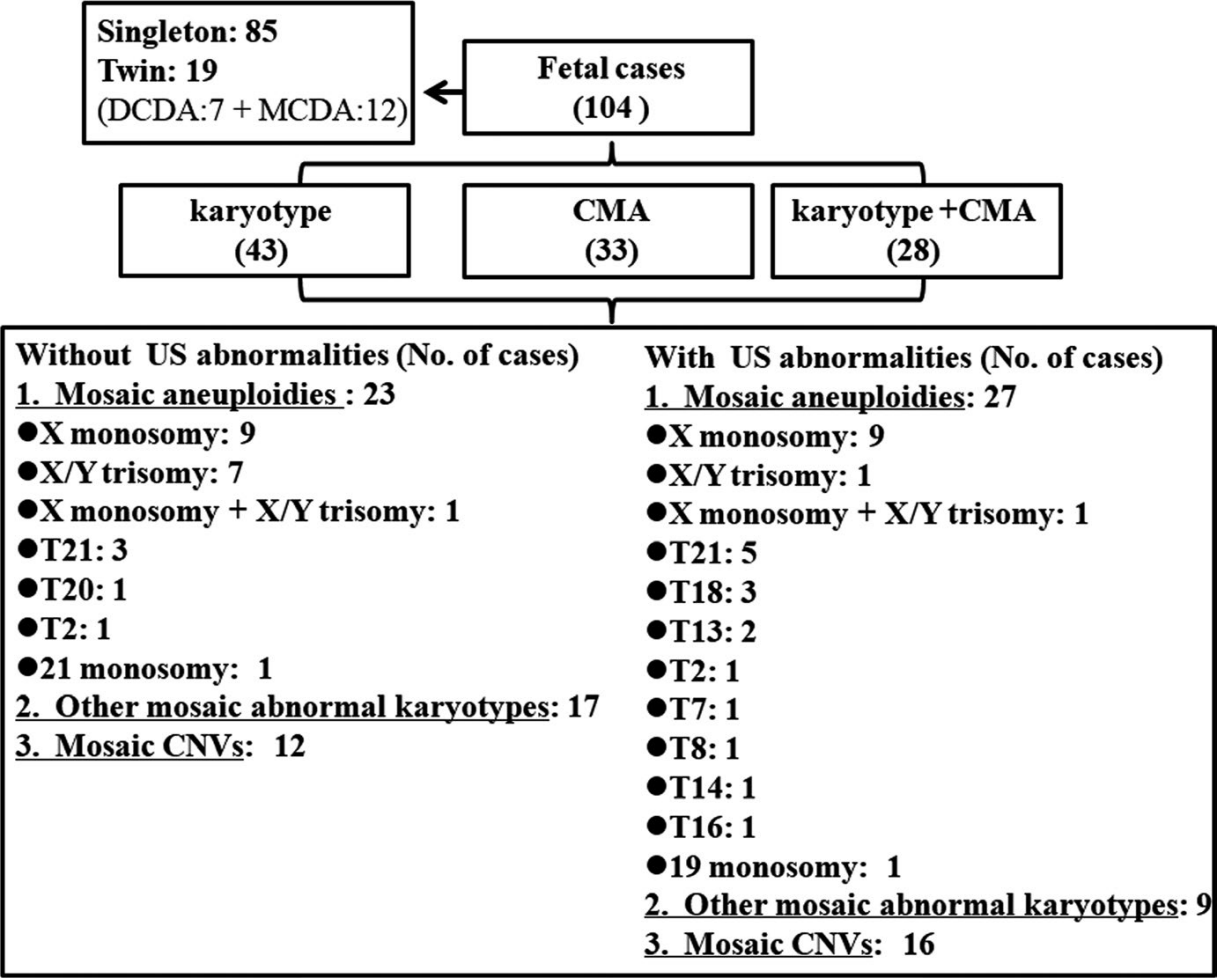
Flow diagram of the chromosomal results in 104 foetal cases with mosaicism. A total of 104 foetal cases were recruited, including 85 singleton pregnancies and 19 pairs of twin pregnancies. Among, forty‐three cases only underwent G‐banding karyotyping, 33 cases only underwent CMA, and 28 cases underwent both karyotyping and CMA. In twin pregnancies, only one foetus was affected by ultrasound anomalies in each pair. In addition, the chromosomal results of the affected foetuses were also reported. Other mosaic abnormal karyotypes refer to abnormal karyotypes detected by G‐banding karyotyping such as isochromosomes and marker chromosomes, not including aneuploidies. CMA, chromosomal microarray analysis; US, ultrasound; No. of, the number of; T, Trisomy; CNVs, copy number variants

We retrospectively reviewed all pregnant women who underwent G‐banding karyotyping and/or chromosomal microarray analysis (CMA) for all indications at Nanfang Hospital from January 2013 to December 2018. There were a total of 5758 karyotyping results and 6066 CMA results. Among them, 104 foetal cases diagnosed with chromosomal mosaicism were selected and further analysed in our study, including 85 singleton pregnancy and 19 twin pregnancies (Figure 1).

Pre‐test counselling was given to all parents, including informing them about the benefits and disadvantages of invasive prenatal diagnosis, the testing process, possible results and limitations of the karyotyping and CMA. Written informed consent to participate and receive invasive prenatal diagnoses was provided by all parents. This study was approved by the Research Ethics Committee of Nanfang hospital and was conducted according to the Declaration of Helsinki principles.

**FIGURE 2:**
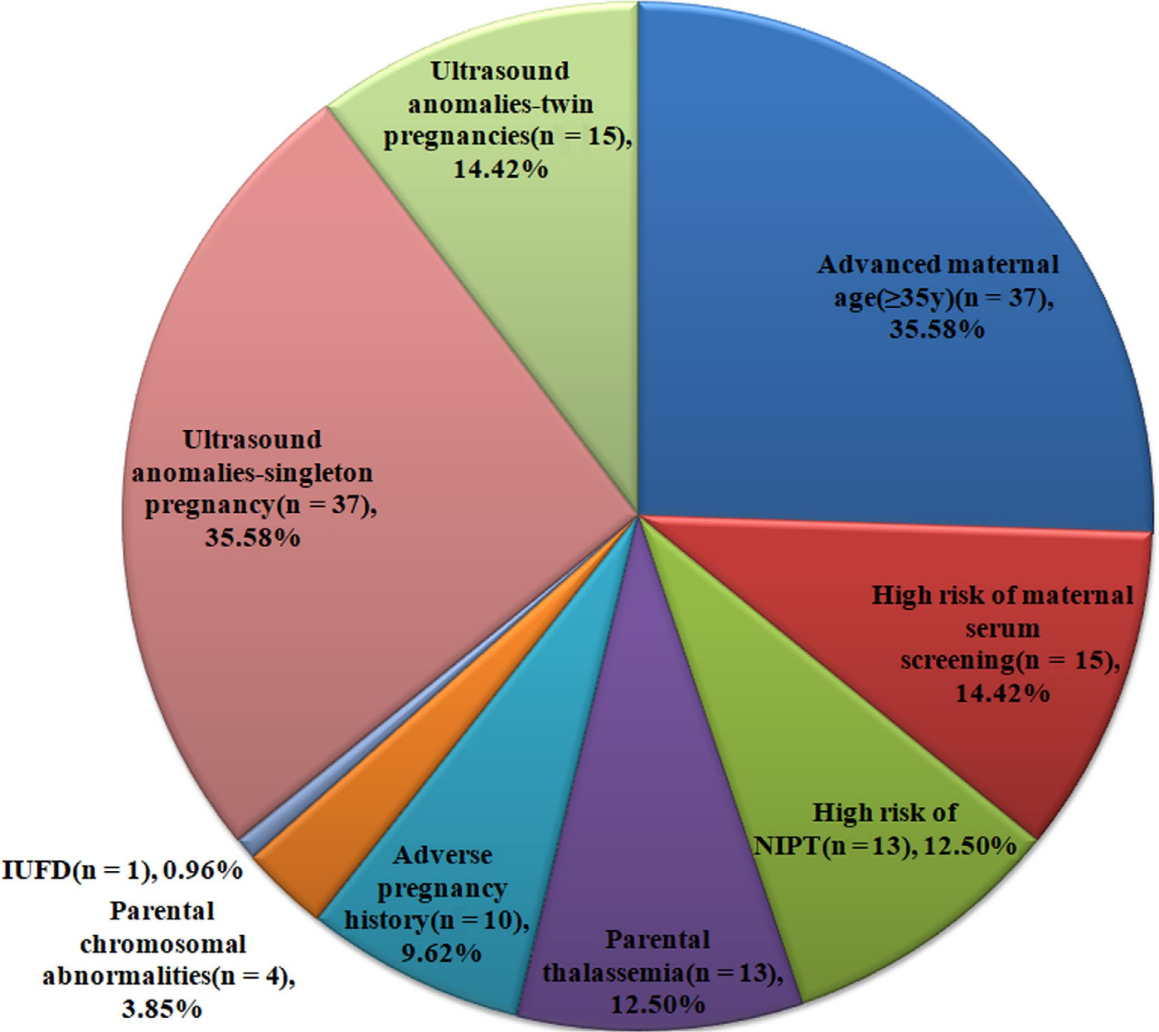
Indications of invasive prenatal diagnosis in foetuses with mosaicism. The sum of the number of cases for different indications exceeds the total number of whole cases (104 cases) because more than one indication may be identified in each case. NIPT, non‐invasive prenatal testing; IUFD, intrauterine foetal death; FGR, foetal growth restriction, n, the number of cases. Parental chromosomal abnormalities include maternal and/or paternal chromosomal abnormalities such as balanced translocation. Ultrasound anomalies include foetal structural defects, soft markers and FGR

Indications for invasive prenatal diagnosis included advanced maternal age, high risk detected by maternal serum screening and non‐invasive prenatal testing, an adverse pregnancy or family history, parental thalassaemia or chromosomal abnormalities, and abnormal ultrasound findings (Figure 2).

Gestational age was determined based on the last menstrual period and an ultrasonogram conducted at 11‐13^+6^ weeks. Foetal growth and anatomy were confirmed by performing an ultrasound examination. Abnormal ultrasound findings included structural defects and soft markers in singleton or twin pregnancies, and foetal growth restriction (FGR) in singleton pregnancies. Soft markers included the absence of a nasal bone, a thickened nuchal translucency or neck fold, ventriculomegaly, echogenic bowel and a shortened long bone.^13^ FGR in a singleton pregnancy is diagnosed when the estimated foetal weight (EFW) is less than the 10th percentile for the gestational age.^14^ The twin pregnancies were classified as monochorionic diamniotic (MCDA) or dichorionic diamniotic (DCDA) according to the ultrasound images at 11‐13^+6^ weeks.^15^

In addition, we also reviewed 80 miscarriage cases with CM detecting by CMA to obtain more information about the potential correlation between CM and its phenotypic effects (Figure S1).

### 2.2 Invasive prenatal diagnosis by karyotyping and CMA

Samples from the chorionic villus, amniotic fluid or cord blood of the foetuses were collected by ultrasound‐guided transabdominal chorionic villus sampling, amniocentesis or cordocentesis. Cells were cultured and prepared for G‐banding karyotyping using standard protocols. Genomic DNA was extracted from the uncultured samples, and then CMA was carried out by using a whole genome‐wide Affymetrix CytoScan HD array (Affymetrix Inc, Santa Clara, CA, USA) according to the manufacturer's operating procedures. The data were analysed by Affymetrix Chromosome Analysis Software Suite (ChAS) 3.0 (Affymetrix, Inc) and annotated with genome version GRCH37/hg19.

Copy number variants (CNVs) were classified as pathogenic, likely pathogenic, variants of uncertain significance (VUSs), likely benign and benign according to the American College of Medical Genetics guidelines.^16^ In addition, microduplications with fragment lengths over 1 Mb, microdeletions with a length over 500 kb, about 30% mosaic CNVs with a length over 5 Mb, and loss of heterozygosity with a length over 10 Mb were all reported in our study.

The theoretical values for the detection of a single duplication or deletion are a log_2_ ratio of above +0.58 or below −1.0, respectively. Mosaicism was determined if the average log_2_ ratio of a chromosome deviated from 0 by more than two standard deviations from 0 up to +0.58 or from 0 down to −1.0.^17^

CNVs were interpreted and analysed by referring to scientific reports and the following public databases: the UCSC (http://genome.ucsc.edu/index.html), database of genomic variants (http://dgv.tcag.ca/dgv/app/home), DECIPHER (https://decipher.sanger.ac.uk/index), ISCA (https://www.iscaconsortium.org/), Online Mendelian Inheritance in Man (http://omim.org/) and ClinGen Dosage Sensitivity Map (http://www.ncbi.nlm.nih.gov/projects/dbvar/clingen/index.shtml).

### 2.3 Statistical analysis

Statistical analyses were performed with SPSS software v22.0 (SPSS Inc, Chicago, IL, USA). The chi‐square test and two independent sample t test were applied to analyse the statistical data. Differences were considered statistically significant when *P* < 0.05.

## 3 RESULTS

### 3.1 Clinical characteristics

A total of 5758 G‐banding karyotyping results and 6066 CMA results from invasive prenatal diagnosis were reviewed, of which 104 foetal cases were identified with CM. Among them, 43 cases underwent karyotyping, 33 cases underwent CMA and 28 cases underwent both karyotyping and CMA (Figure 1). The indications for invasive prenatal diagnosis of the 104 prenatal cases with CM are listed in Figure 2. Ultrasound anomalies were found in 50% (52/104) cases, including 37 singleton pregnancies and 15 twin pregnancies. Advanced maternal age was the most common indication for patients with CM (35.58%, 37/104) after excluding ultrasound anomalies.

The clinical characteristics of the patients were further investigated based on cases complicated with or without ultrasound anomalies as shown in Table 1. The mean maternal ages were 30.38 ± 4.86 and 33.63 ± 5.03 years in patients with and without ultrasound anomalies, respectively. The number of patients older than 35 years was higher in cases without ultrasound anomalies (46.15%, 24/52) than those in cases with ultrasound anomalies (23.08%, 12/52). The mean gestational ages for undergoing invasive prenatal diagnosis were 19.48 ± 4.32 and 22.83 ± 6.05 weeks in patients without and with ultrasound anomalies, respectively.

**TABLE 1:** Clinical characteristics in cases with and without ultrasound anomalies

Characteristics	US anomalies		*P* vaule^a^
	No (n = 52)	Yes (n = 52)	
MA, years			
Mean ± SD	33.63 ± 5.03	30.38 ± 4.86	–
<35; n(%)	28 (53.85%)	40 (76.92%)	0.023*
≥35; n(%)	24 (46.15%)	12 (23.08%)	0.023*
GA, weeks			
Mean ± SD	19.48 ± 4.32	22.83 ± 6.05	–
Specimen source			
CVS; n (%)	7 (13.46%)	4 (7.69%)	–
AF; n (%)	30 (57.69%)	25 (48.08%)	–
CB; n(%)	15 (28.85%)	23 (44.23%)	–
Overall classification			
Mosaic aneuploidies; n(%)	23 (44.23%)	27 (51.92%)	0.556
Mosaic other abnormal karyotypes^b^; n(%)	17 (32.69%)	9 (17.31%)	0.112
Mosaic CNVs; n(%)	12 (23.08%)	16 (30.77%)	0.508

Note

Chi‐square tests were used and all *P* values are two‐sided.*P* value^a^: comparisons were performed between cases with and without ultrasound anomalies (*P < 0.05). Mosaic other abnormal karyotypes^b^ refer to abnormal karyotypes detected by G‐banding karyotyping such as isochromosomes, marker chromosomes, but not including aneuploidies.

Abbreviations: AF, amniotic fluid; CB, cord blood; CNVs, copy number variantsCVS, chorionic villus sampling; US, ultrasound.

In all patients without ultrasound anomalies, 7 cases were detected from chorionic villus sampling (CVS), 30 from amniotic fluid (AF) and 15 from cord blood (CB). Among overall classification, the rate of mosaic aneuploidies was 44.23% (23/52), the rate of other abnormal karyotypes was 32.69% (17/52), and that of mosaic CNVs was 23.08% (12/52). In cases with ultrasound anomalies, 4 cases were detected from CVS, 25 from AF and 23 from CB. In overall classification, the rate of mosaic aneuploidies was 51.92% (27/52), that of other abnormal karyotypes was 17.31% (9/52), and that of mosaic CNVs was 30.77% (16/52).

### 3.2 Ultrasound anomalies

Fifty‐two foetal cases were affected by ultrasound anomalies among 104 cases, including 37 singleton pregnancies and 15 twin pregnancies. In the twin pregnancies, only one foetus was affected in each pair. The detailed ultrasound anomalies are shown in Table 2.

**TABLE 2:** Ultrasound anomalies* in foetuses with mosaicism

US anomalies	Singleton pregnancy No. of cases (%^a^) (total: 37)	Twin pregnancies No. of cases (%^a^) (total: 15)
FGR	5 (13.51%)	–
Structural defects	25 (67.57%)	15 (100%)
Isolated in single system	19 (51.35%)	13 (86.67%)
Non‐isolated in multiple system	6 (16.22%)	2 (13.33%)
Structural defects distributions		
Cardiovascular	10 (27.03%)	4 (26.67%)
Urogenital	4(10.81%)	0
Gastrointestinal	4 (10.81%)	0
Hydrops fetalis	3 (8.11%)	3 (20.00%)
Central nervous	2 (5.41%)	2 (13.33%)
Musculoskeletal	2 (5.41%)	0
Faciocervical	1 (2.70%)	0
Cystic hygroma	1 (2.70%)	4 (26.67%)
other^b^	2 (5.41%)	1 (6.67%)
Soft markers	9 (24.32%)	2 (13.33%)
Single	7 (18.92%)	2 (13.33%)
Multiple	2 (5.41%)	0
Soft markers distributions		
Thickened NT/NF	7 (18.92%)	0
NB absence/ dysplasia	3 (8.11%)	0
Shortened long bone	1 (2.70%)	1 (6.67%)
Ventriculomegaly	0	1 (6.67%)

Note

The sum of different ultrasound abnormalities exceeds the total number of singleton or twin pregnancies due to more than one ultrasound anomaly detected in one case. US anomalies* include foetal structural defects, soft markers and FGR. (%^a^): the percentages listed in Table 3 were used to calculate the detailed ratio of different types of ultrasound anomalies in cases with ultrasound phenotypes, but not used to calculate the detection rates of ultrasound anomalies in all 104 cases. other^b^ refer to other structural defects such as isomerism syndrome, excluding structural defects listed in Table 3.

Abbreviations: FGR, foetal growth restriction; NB, nasal bone; NF, neck fold; NT, nuchal translucency; US, ultrasound.

In singleton pregnancies, FGR was identified in 5 foetuses (ratio = 13.51%, 5/37), structural defects were found in 25 foetuses (ratio = 67.57%, 25/37), and soft markers were found in 9 foetuses (ratio = 24.32%, 9/37). Among these, most of the structural defects and soft markers were isolated defects in one system (ratio = 51.35%, 19/37) or a single soft marker (ratio = 18.92%, 7/37). Cardiovascular defects were the most frequent structural defect (ratio = 27.03%, 10/37), followed by urogenital defects (ratio = 10.81%, 4/37) and gastrointestinal defects (ratio = 10.81%, 4/37). A thickened nuchal translucency or nuchal fold was found to be the most common soft marker (ratio = 18.92%, 7/37). In 15 twin pregnancies, the structural defects were all detected in only one foetus of the twin pair, among which the majority only had an isolated defect in one system (ratio = 86.67%, 13/15). Cardiovascular defects (ratio = 26.67%, 4/15) and cystic hygromas (ratio = 26.67%, 4/15) were the most common defects, while 2 soft markers as a single marker were detected in two different foetuses.

### 3.3 Chromosomal mosaicism

**FIGURE 3:**
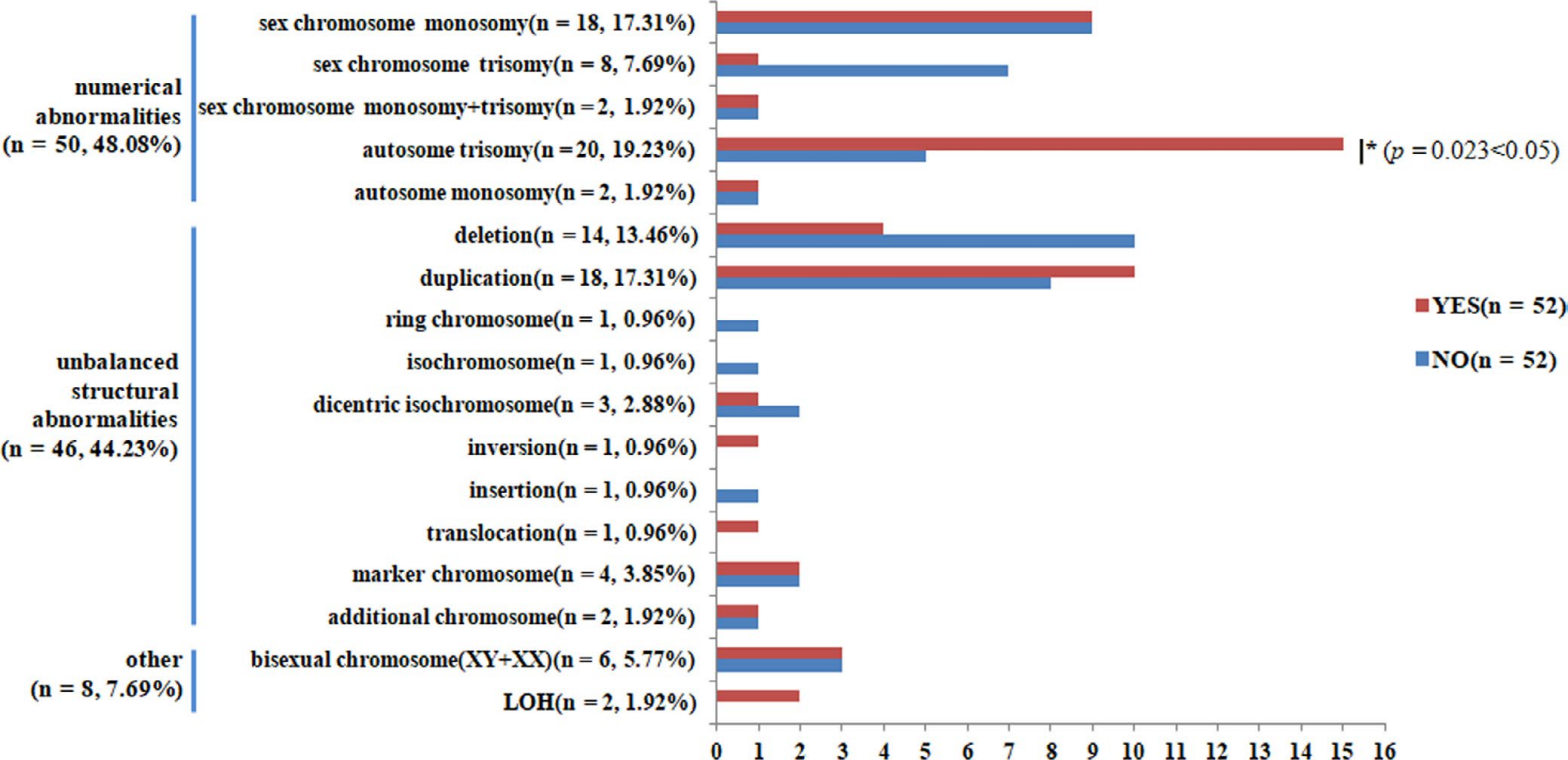
Incidences of the detailed types of mosaicisms detected in this study. Comparisons for mosaic types were performed between cases with and without ultrasound phenotypes, and Chi‐square tests were used and all *P* values are two‐sided. Mosaic chromosomal abnormalities were divided into three types: numerical abnormalities, unbalanced structural abnormalities and other. The rate of autosomal trisomy in cases with ultrasound anomalies was higher than that in cases without (*P = 0.023 < 0.05). YES, cases with ultrasound anomalies; NO, cases without ultrasound anomalies; LOH, loss of heterozygosity; n, the number of cases

Figure 3 summarizes the distribution of detailed mosaic types. Overall, numerical abnormalities were identified in 48.08% (50/104) of cases, of which mosaic autosomal trisomy was the most common type (19.23%, 20/104), followed by sex chromosomal monosomy‐X monosomy (17.31%, 18/104). Meanwhile, unbalanced structural abnormalities were found in 44.23% (46/104) cases, among which duplications (17.31%, 18/104) and deletions (13.46%, 14/104) were the most frequent types. Other chromosomal mosaicisms were also detected in 8 cases, covering 6 cases with bisexual chromosomes (XY/XX) and 2 cases with mosaic LOH. Detailed information about the mosaic pathogenic CNVs and chromosomal aberrations are shown in Table S1 and Table S2.

To explore the correlation of CM and its phenotypic effects, we further compared the differences of mosaic types and mosaic fractions between cases with and without ultrasound anomalies. First, the incidence of autosomal trisomy in cases with ultrasound anomalies (28.85%, 15/52) was much higher than that in cases without (9.62%, 5/52) (*P* = 0.023 < 0.05) (Figure 3). In addition, 89.04% (65/73) of miscarriage cases were diagnosed as mosaic autosomal trisomy, excluding one case with 48, XXY, +9 (Figure S1). The highest detection rate of mosaic autosomal trisomy was found in miscarriage cases, followed by foetuses with ultrasound anomalies, and finally cases without phenotypes (*P* = 0.000 < 0.05). In cases of mosaic unbalanced structural abnormalities and other chromosomal mosaicisms, no significant differences were identified between foetuses with and without ultrasound phenotypes (*P* > 0.05) (Figure 3). Second, in mosaic fractions, there were also no differences detected in the mosaic fractions whether it was based on the specimen sources or the overall classification (Table S3). We then investigated mosaic fractions of aneuploidies and pathogenic CNVs both detected in foetuses with and without phenotypes as shown in Table S4. The most common mosaic aneuploidy in the two groups was 45, X/46, XN, while no difference was found in its mosaic fraction (*P* = 0.090 > 0.05). Similar results were also detected in other aneuploidies and for pathogenic CNVs (Table S4).

### 3.4 Discordant chromosomal mosaicism

In our cohort, discordant mosaic results were observed in 16 of the 104 cases due to different specimen sources or different testing technologies (15.38%, 16/104), including 4 with ultrasound phenotypes and 12 without. Excluding the foetuses only with different mosaic fractions, 9 cases with truly discordant CM were identified, as listed in Table 3. Eight discordant mosaicisms were detected from different specimen sources, of which 7 cases were derived from the AF and CB, and 1 from the CVS and AF.

**TABLE 3:** Discordant chromosomal results^a^ in foetuses with mosaicism

No.	MA	indication(s)	1st results	2nd results
13	31	high risk of MSS for T21	**AF**: 45,X,1qh+[18]/46,X,i(Y)?[12]	**CB**: 46,X,idic(Y)(q11.23)[34]/45,X[12]/47,X,idic(Y)(q11.23)×2[4]
30	37	advanced MA; parental thalassaemia	**AF**: 47,XX,+i(12)(p10)[9]/46,XX[191] 12p13.33q11(173789‐37869107) ×3(pCNV) (CMA)	**CB**: 47,XX,+del(12)(q12)[2]/46,XX[48] 12p13.33p11.1(173786‐34835837)×3(pCNV) (CMA)
97	39	advanced MA	**AF**:47,XY,+del(15)(q15)?[75]/46,XY[33]	**CB**: 47,XY,+mar[43]/46,XY[24] 15q11.2q14(22770421‐34488168) ×4(pCNV) (CMA)
98	40	advanced MA	**AF**:46,XN,1qh+,?ins(10;1)(q22;q44q21)[15]/46,XN,1qh+[89]	**CB**: normal(CMA)
99	30	maternal balanced translocation	**CVS**: 46,XN,del(1)(q11)[7]/46,XN[14]	**AF**: normal(CMA)
100	28	US: foetal thickened NT	**AF**:46,XX[63]/46,XX,der(13;13)(q10;q10),+mar[4]	**CB**: normal(CMA)
101	43	advanced MA	**AF**: 47,XN,+20[13]/46,XN[80]	**CB**: normal(CMA)
102	35	advanced MA	**AF**: 46,XN,+del(1)(q12)[13]/46,XN[37]	**CB**: normal(CMA)
87	37	F1: normal F2: hydrops fetalis, ventriculomegaly. advanced MA.	**AF**: F1: 45,X[26]/46X,psu idic(Y)(p11.3)[5] F1: Xp22.33(168546‐1772773)×1(pCNV) (CMA) 16p13.3(7204004‐7317958)×1(VUSs) (CMA) F2: Xp22.33(168546‐1771538)×1(pCNV) (CMA) 14q11.2(22289605‐22995570)×1(VUSs) (CMA) 16p13.3(7203952‐7317958)×1(VUSs) (CMA)	‐

Note

Discordant chromosomal results^a^: discordant mosaic chromosomal results in the same foetus are listed in this table, not including the cases with the same mosaic chromosomes but different mosaic fractions. Case 87: this case was a monochorionic diamniotic twin pregnancies, of that foetus 1(normal) underwent karyotyping and CMA, while foetus 2 (hydrops fetalis) underwent CMA. The discordant results were detected between G‐banding karyotyping and CMA.

Abbreviations: AF, amniotic fluid; CB, cord blood; CMA, chromosomal microarray analysis; CVS, chorionic villus sampling; MA, maternal age; MSS, maternal serum screening; No., number; pCNVs, pathogenic copy number variants; T, trisomy; US, ultrasound; VUSs, variants of uncertain significant.

In cases 13, 30 and 97, different karyotypes were detected in the AF and CB. In cases 98, 100, 101 and 102, abnormal karyotypes were found in the AF, while the CMA results from the CB were normal. Notably, in 12 mosaicisms detected by CVS, 2 cases also underwent amniocentesis to differentiate confined placental mosaicism (CPM) from true foetal mosaicism (TFM) (case 99 in Table 3 and case 2 in Table S5). Case 2 was verified as TFM for Turner syndrome, while case 99 was confirmed as CPM. In addition, in the MCDA twin pair of case 87, a mosaicism that combined X monosomy and a pseudo dicentric isochromosome was found by karyotyping in foetus 1 without ultrasound anomalies, while CMA only detected a 1.604 Mb pathogenic CNV associated with Leri‐Weill dyschondrostosis and a 114 kb VUS in the same foetus.

## 4 DISCUSSION

Unlike general chromosomal abnormalities, CM may be defined as a coexistence of normal cells and abnormal cells or multiple types of abnormal cells. The phenotypic effects in patients with CM are considered to be complicated and indistinct. Especially for foetuses in utero, it is challenging to provide objective data to determine the risk of true foetal involvement and the clinical relevance.

In our data, only 50% of foetal cases with CM were diagnosed with ultrasound anomalies, while the other 50% had no ultrasound phenotypes. Lund ICB *et al* reported that a thickened NT was only identified in 8.6% of foetuses with mosaic whole chromosomes and 29.6% with mosaic CNVs.^10^ Malvestiti et al showed that no major foetal anomalies were found by prenatal ultrasound scans in 13% TFM cases.^4^ However, in their follow‐up, deep plantar furrows and a malpositioned fourth toe were observed in a neonate with mosaic trisomy 8 at birth.^4^ Thus, whether these 50% no‐phenotype foetuses in our study are truly normal requires long‐term follow‐up after birth.

Ultrasound anomalies in foetuses with CM seem variable, and no evidence was found that a CM may account for some ultrasound anomalies. Approximately 50% of our CM carriers had ultrasound anomalies, of which isolated structural defects in one system and single soft markers were the most common findings in both singleton and twin pregnancies. In a study with 100 CVS samples, 5 mosaic chromosomal aberrations were detected, of which two cases both showed an isolated but complex structural defect in one system, including a hypoplastic right ventricle of the heart in one foetus and corpus callosum and hypoplasia of the cerebellar vermis in another foetus.^18^ This suggested that CM foetuses with phenotypes are probably not associated with multiple structural defects or soft markers, but instead are often detected as isolated ultrasound anomalies.

In addition, 5 foetuses with CM were diagnosed as FGR and their samples were all from cord blood, excluding the possibility of CPM. A previous study suggested that mosaicisms from CVS should be further verified for TFM by AF or CB due to the presence of fetoplacental discrepancies.^19^ However, although most pregnancies diagnosed with CPM are deemed to have good postnatal outcomes but an impaired placenta may provide insufficient support for the pregnancy, leading to foetal complications such as FGR or other adverse outcomes.^20^, ^21^ Therefore, continuous monitoring of foetal growth during pregnancy is important to prevent adverse complications for foetuses with TFM and CPM.

The incidences of CM were compared between foetuses with and without phenotypes. Among these, the incidence of mosaic autosomal trisomy was higher in phenotypic foetuses than that in no‐phenotypic foetuses. Interestingly, gradually increased detection rates of mosaic autosomal trisomy were found from data in foetuses without ultrasound phenotypes to cases with phenotypes and to miscarriage samples. We speculated that most of the mosaic autosomal trisomy induced by meiotic or mitotic non‐disjunction error remains confined to the placenta because they are almost universally lethal or pathogenic for foetuses. For this reason, the incidence of mosaic autosomal trisomy in miscarriage samples was the highest among all prenatal cases.^22^

Another critical factor possibly impacting the phenotypic effect of CM is mosaic fractions. In our study, even for the same CM detected in two groups, mosaic fractions displayed no difference, which is not very consistent with previous reports. In cancer research, high‐level mosaicism is detected in patients with bilateral and unilateral retinoblastoma, while low‐level mosaicism is only detected in patients with unilateral retinoblastoma, suggesting the level of mosaicism is correlated with clinical parameters such as disease phenotype.^23^ Whereas in a child with a mosaic 12p partial isochromosome, different tissues were all detected to have a high percentage of aneuploid cells, but the patient presented with dyschromia as the sole manifestation.^24^

Similarly to our results in prenatal cases, Vogel et al showed that 2 foetuses with major structural defects had only 10%–30% mosaicism, while 3 foetuses without ultrasound anomalies had 50%–60%, 60‐70% or 10% mosaicism, respectively.^18^ This observation reveals that the severity of the phenotypes cannot be simply determined by mosaic fractions. In addition, considering the differences and limitations of technical conditions and bioinformatics analysis in each genetic laboratory, it will be increasingly important to establish thresholds that are critical to define low‐ or high‐level mosaicism by using more accurate techniques.

Discordant mosaicisms were identified from different specimens or testing methods in our study. When amniocentesis or cordocentesis revealed a normal result, CPM may have accounted for the majority of CM from CVS. Previous data considered most of mosaicisms from CVS to be unreliable due to TFM being only detected in 4%–28.15% of cases.^4^, ^25^, ^26^ Battaglia et al indicated the even when all samples were from CVS, discordant results were still found between short‐term and long‐term culture methods.^25^ In addition, AF and CB represent different foetal tissues, of which AF is considered to be the optimal specimen for foetal confirmation because it includes cells mainly from foetal anatomical districts including the urogenital tract, the respiratory tract, and the epithelial systems, representing different embryological layers.^27^ However, discordant results between AF and CB of our data suggested the complexity of mosaicisms from different tissues may be more common than we realize. Chen et al revealed a foetus with Pallister‐Killian syndrome, in that different mosaic tetrasomy 12p was identified from amniotic fluid, skin, placenta and cord blood by karyotyping, FISH, QF‐PCR and array‐CGH.^28^ Their results showed that CB and placentas were prone to a negative result when compared with AF, and array‐CGH on uncultured CB or FISH on cultured CB may be better to use for prenatal diagnosis. Others hold the opposite view that mosaicism in AF requires further cordocentesis for confirmation, and that the pregnancy is safe when a normal result is identified in CB.^29^ These findings demonstrate that increased risk in genetic counselling is due to discordant CM from different specimens or testing methods. It is highly recommended to use more comprehensive assays such as a combination of CMA, FISH and karyotyping to detect mosaicism in AF and CB before any irreversible decision is made in regard to the pregnancy.

There are a few drawbacks in our study. The first is our uncompleted long‐term follow‐up may lead to information loss for postnatal phenotypes. The second is the presence of selection bias in this retrospective analysis. The incidence or detection rate of CM may be higher in our data than in the general population. The third is the confirmation in AF or CB was performed in only a small proportion of cases from CVS, directly causing failure to distinguish CPM or TFM in the rest of the cases.

In summary, the incidence and clinical characteristics of CM were investigated in foetuses with and without ultrasound phenotypes by karyotyping and CMA, providing valuable information for genetic counselling and management of prenatal mosaic cases. Greater caution should be used in prenatal counselling, and more comprehensive assays involving serial ultrasound examinations, different specimens or testing methods verifications and effective follow‐up should be applied.

## CONFLICT OF INTEREST

The authors declare that they have no conflict of interest.

## AUTHOR CONTRIBUTIONS

**Yi Zhang:** Funding acquisition (lead); Investigation (equal); Methodology (equal); Software (equal); Writing‐original draft (lead); Writing‐review & editing (lead). **Mei Zhong:** Conceptualization (equal); Data curation (lead); Project administration (lead); Supervision (equal). **Dezhong Zheng:** Conceptualization (equal); Formal analysis (equal); Investigation (equal); Methodology (equal); Software (equal); Supervision (equal); Writing‐review & editing (equal).

## Supporting information

Fig S1

Table S1‐S5

## Data Availability

The datasets analysed in this study are available from the corresponding author on reasonable request.

## References

[1] van Echten‐Arends J , Mastenbroek S , Sikkema‐Raddatz B , et al. Chromosomal mosaicism in human preimplantation embryos: a systematic review. Hum Reprod Update. 2011;17:620‐627. 21531753 10.1093/humupd/dmr014

[2] Grati FR . Chromosomal Mosaicism in Human Feto‐Placental Development: Implications for Prenatal Diagnosis. J Clin Med. 2014;3:809‐837. 26237479 10.3390/jcm3030809 PMC4449651

[3] Taylor TH , Gitlin SA , Patrick JL , et al. The origin, mechanisms, incidence and clinical consequences of chromosomal mosaicism in humans. Hum Reprod Update. 2014;20:571‐581. 24667481 10.1093/humupd/dmu016

[4] Malvestiti F , Agrati C , Grimi B , et al. Interpreting mosaicism in chorionic villi: results of a monocentric series of 1001 mosaics in chorionic villi with follow‐up amniocentesis. Prenat Diagn. 2015;35:1117‐1127. 26213308 10.1002/pd.4656

[5] Chen CP , Hsu CY , Chern SR , et al. Prenatal diagnosis of mosaic trisomy 8 by amniocentesis in a fetus with ventriculomegaly and dysgenesis of the corpus callosum. Taiwan J Obstet Gynecol. 2020;59:127‐129. 32039780 10.1016/j.tjog.2019.11.020

[6] Masoudzadeh N , Teimourian S . Comparison of quantitative fluorescent polymerase chain reaction and karyotype analysis for prenatal screening of chromosomal aneuploidies in 270 amniotic fluid samples. J Perinat Med. 2019;47:631‐636. 31194688 10.1515/jpm-2019-0069

[7] Shubina J , Trofimov DY , Barkov IY , et al. In silico size selection is effective in reducing false positive NIPS cases of monosomy X that are due to maternal mosaic monosomy X. Prenat Diagn. 2017;37:1305‐1310. 29110322 10.1002/pd.5178

[8] Mellis R , Chandler N , Jenkins L , et al. The role of sonographic phenotyping in delivering an efficient non‐invasive prenatal diagnosis (NIPD) service for FGFR3‐related skeletal dysplasias. Prenat Diagn. 2020;40:785–791. 32227640 10.1002/pd.5687

[9] Stern S , Hacohen N , Meiner V , et al. Universal chromosomal microarray analysis reveals high proportion of copy number variants in low risk pregnancies. Ultrasound Obstet Gynecol. 2020 Mar 23. 10.1002/uog.22026 32202684

[10] Lund ICB , Becher N , Christensen R , et al. Prevalence of mosaicism in uncultured chorionic villus samples after chromosomal microarray and clinical outcome in pregnancies affected by confined placental mosaicism. Prenat Diagn. 2020;40:244‐259. 31769052 10.1002/pd.5584

[11] Daniel A , Wu Z , Darmanian A , et al. Issues arising from the prenatal diagnosis of some rare trisomy mosaics–the importance of cryptic fetal mosaicism. Prenat Diagn. 2004;24:524‐536. 15300743 10.1002/pd.936

[12] Nagamatsu T , Kamei Y , Yamashita T , et al. Placental abnormalities detected by ultrasonography in a case of confined placental mosaicism for trisomy 2 with severe fetal growth restriction. J Obstet Gynaecol Res. 2014;40:279‐283. 24033883 10.1111/jog.12145

[13] Reddy UM , Abuhamad AZ , Levine D , et al. Fetal imaging: Executive summary of a Joint Eunice Kennedy Shriver National Institute of Child Health and Human Development, Society for Maternal‐Fetal Medicine, American Institute of Ultrasound in Medicine, American College of Obstetricians and Gynecologists, American College of Radiology, Society for Pediatric Radiology, and Society of Radiologists in Ultrasound Fetal Imaging Workshop. Am J Obstet Gynecol. 2014;210:387‐397. 24793721 10.1016/j.ajog.2014.02.028

[14] Martins JG , Biggio JR , Abuhamad A . Society for Maternal‐Fetal Medicine (SMFM) Consult Series #52: Diagnosis and Management of Fetal Growth Restriction. Am J Obstet Gynecol. 2020;223:B2–B17. 10.1016/j.ajog.2020.05.010 32407785

[15] Emery SP , Bahtiyar MO , Moise KJ . The North American Fetal Therapy Network Consensus Statement: Management of Complicated Monochorionic Gestations. Obstet Gynecol. 2015;126:575‐584. 26244534 10.1097/AOG.0000000000000994

[16] Richards S , Aziz N , Bale S , et al. Standards and guidelines for the interpretation of sequence variants: a joint consensus recommendation of the American College of Medical Genetics and Genomics and the Association for Molecular Pathology. Genet Med. 2015;17:405‐424. 25741868 10.1038/gim.2015.30 PMC4544753

[17] Carey L , Scott F , Murphy K , et al. Prenatal diagnosis of chromosomal mosaicism in over 1600 cases using array comparative genomic hybridization as a first line test. Prenat Diagn. 2014;34:478‐486. 24453008 10.1002/pd.4332

[18] Vogel I , Vestergaard EM , Lildballe DL , et al. Placental mosaicism in the era of chromosomal microarrays. Eur J Med Genet. 2020;63:103778. 31580923 10.1016/j.ejmg.2019.103778

[19] Grati FR , Bajaj K , Zanatta V , et al. Implications of fetoplacental mosaicism on cell‐free DNA testing for sex chromosome aneuploidies. Prenat Diagn. 2017;37:1017‐1027. 28801976 10.1002/pd.5138

[20] Grati FR , Ferreira J , Benn P , et al. Outcomes in pregnancies with a confined placental mosaicism and implications for prenatal screening using cell‐free DNA. Genet Med. 2020;22:309‐316. 31391534 10.1038/s41436-019-0630-y

[21] Cheng HH , Ma GC , Tsai CC , et al. Confined placental mosaicism of double trisomies 9 and 21: discrepancy between non‐invasive prenatal testing, chorionic villus sampling and postnatal confirmation. Ultrasound Obstet Gynecol. 2016;48:251‐253. 26663618 10.1002/uog.15840

[22] Dhillon RK , Hillman SC , Morris RK , et al. Additional information from chromosomal microarray analysis (CMA) over conventional karyotyping when diagnosing chromosomal abnormalities in miscarriage: a systematic review and meta‐analysis. BJOG. 2014;121:11‐21. 10.1111/1471-0528.12382 23859082

[23] Rodríguez‐Martín C , Robledo C , Gómez‐Mariano G , et al. Frequency of low‐level and high‐level mosaicism in sporadic retinoblastoma: genotype‐phenotype relationships. J Hum Genet. 2020;65:165‐174. 31772335 10.1038/s10038-019-0696-z

[24] Alesi V , Dentici ML , Restaldi F , et al. Unclassifiable pattern of hypopigmentation in a patient with mosaic partial 12p tetrasomy without Pallister‐Killian syndrome. Am J Med Genet A. 2017;173:1943‐1946. 28489314 10.1002/ajmg.a.38269

[25] Battaglia P , Baroncini A , Mattarozzi A , et al. Cytogenetic follow‐up of chromosomal mosaicism detected in first‐trimester prenatal diagnosis. Prenat Diagn. 2014;34:739‐747. 24633594 10.1002/pd.4358

[26] Gu S , Jernegan M , Van den Veyver IB , et al. Chromosomal microarray analysis on uncultured chorionic villus sampling can be complicated by confined placental mosaicism for aneuploidy and microdeletions. Prenat Diagn. 2018;38:858‐865. 30094853 10.1002/pd.5342

[27] Cremer M , Treiss I , Cremer T , et al. Characterization of cells of amniotic fluids by immunological identification of intermediate‐sized filaments: presence of cells of different tissue origin. Hum Genet. 1981;59:373‐379. 6174407 10.1007/BF00295475

[28] Chen CP , Peng CR , Chern SR , et al. Interphase fluorescence in situ hybridization characterization of mosaicism using uncultured amniocytes and cultured stimulated cord blood lymphocytes in prenatally detected Pallister‐Killian syndrome. Taiwan J Obstet Gynecol. 2014;53:566‐571. 25510702 10.1016/j.tjog.2014.09.004

[29] Sun Y , Zhang P , Zhang N , et al. Cytogenetic analysis of 3387 umbilical cord blood in pregnant women at high risk for chromosomal abnormalities. Mol Cytogenet. 2020;13:2. 31998409 10.1186/s13039-020-0469-6 PMC6979326

