# Supplementary figures and images for: Chromosomal mosaicism detected by karyotyping and chromosomal microarray analysis in prenatal diagnosis
Source: J Cell Mol Med. 2020 Nov 17;25(1):358–66. doi: 10.1111/jcmm.16080 (PMC7810963; doi:10.1111/jcmm.16080)

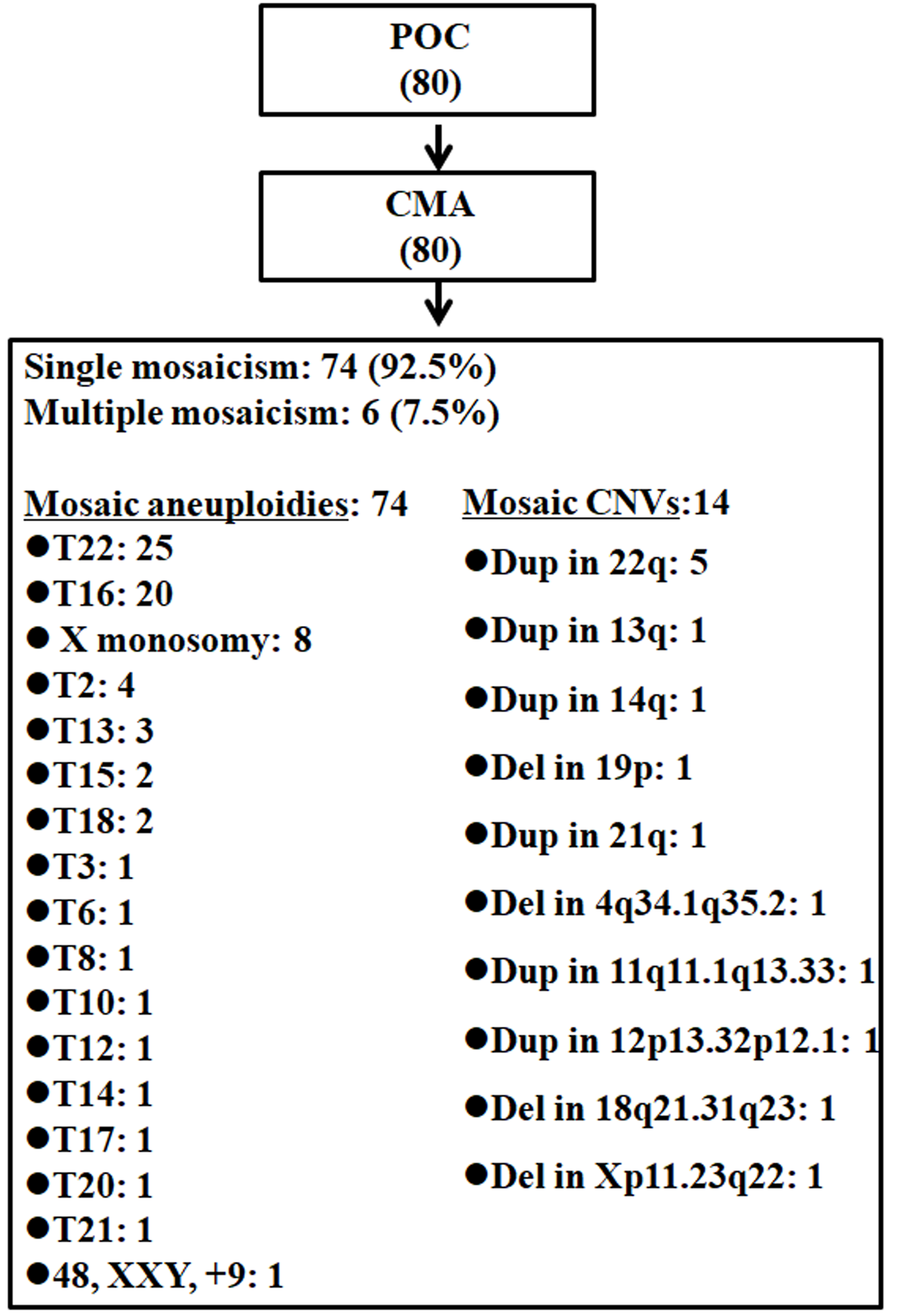

Supplement: Supplementary file 1 — Fig S1 [file JCMM-25-358-s001.tif]
