# Supplementary material for: Chromosomal mosaicism detected by karyotyping and chromosomal microarray analysis in prenatal diagnosis
Source: J Cell Mol Med. 2020 Nov 17;25(1):358–66. doi: 10.1111/jcmm.16080 (PMC7810963; doi:10.1111/jcmm.16080)
Supplement: Supplementary file 2 — Table S1‐S5 [file JCMM-25-358-s002.docx]

Table S1. Pathogenic copy number variations detected in fetuses with and without ultrasound anomalies

| No. | MA  years | GA  weeks | specimen source | indication(s) | karyotype | CMA |
| --- | --- | --- | --- | --- | --- | --- |
| **cases without US anomalies** | | | | |  |  |
| 70 | 24 | 26 | CB | the history of hydrops fetalis | - | 22q11.21(18648866- 21465659)×3, 2.817 Mb  22q11.1q11.22(17577950-22740462)×2.47, 5.163 Mb |
| 97 | 39 | 22^+5^ | AF/CB | advanced MA | AF:47,XY,+del(15)(q15)?[75]/46,XY[33]  CB: 47,XY,+mar[43]/46,XY[24] | CB: 15q11.2q14(22770421-34488168) ×4, 11.718 Mb |
| 94 | 30 | 18^+2^ | AF | high risk of NIPT for chr X | - | Xpterp11.21(168546-55476636) ×1, 55.308 Mb  Xp11.21qter (55548945-155198375) ×2.71, 99.65 Mb |
| 55 | 35 | 19^+5^ | AF | high risk of NIPT for chr18; advanced MA; adverse pregnancy history | - | 18p11.32p11.21(190071-14553958) ×2.26, 14.364Mb |
| 51 | 36 | 12 | CVS | advanced MA; adverse pregnancy history | - | 22q11.1q13.33(16888889-51197766) ×2.77, 34.309Mb |
| 68 | 38 | 17 | AF | high risk of NIPT for chr 2; advanced MA | - | 2p25.1p23.3(10582206-26367503) ×1.74, 15.785Mb |
| 63 | 30 | 17^+1^ | AF | IUFD | - | Xp11.23q22.1(49011035-99716478) ×1.74-1.78, 50.705Mb |
| 30 | 37 | 29 | AF/CB | parental thalassemia; advanced MA | AF: 47,XX,+i(12)(p10)[9]/46,XX[191]  CB: 47,XX,+del(12)(q12)[2]/46,XX[48] | AF: 12p13.33q11(173789-37869107) ×3, 37.695Mb  CB: 12p13.33p11.1(173786-34835837)×3, 34.66 Mb |
| **cases with US anomalies** | | | | |  |  |
| 77 | 26 | 26^+5^ | CB | US: FGR | 46,XN,15ph+ | 22q11.21(18645353-21465659) ×3, 2.820Mb  22q11.1q11.23(16888899-23554942) ×2.57, 6.66Mb |
| 86 | 29 | 17^+6^ | AF | US: fetal thickened NT | 46,X,+mar[32]/45,X[8] | Xp22.33p11.22(168546-54050826) ×1, 53.882 Mb  Xq21.1q28(76732460-155233731) ×1, 78.478 Mb  Xp11.22q21.1(54113305-76723034) ×1.5-2.0, 22.61 Mb  (Y) ×0 |
| 66 | 38 | 28 | CB | US: fetal intestinal duplication; advanced MA | - | 2q11.2q37.3(101169840-242783384) ×2.2-2.3, 141.614 Mb |
| 58 | 43 | 18^+3^ | AF | US: fetal thickened NT; advanced MA | - | Xp11.22q13.1(53858865-68538091) ×2.22, 14.679Mb |
| 74 | 29 | 27^+5^ | CB | US: fetal NB dysplasia  parental thalassemia | - | 10q23.33q26.3 (95248243-135427143) ×2.37, 40.179 Mb |

No., number; MA, maternal age; GA, gestational age; CVS, chorionic villus sampling; AF, amniotic fluid; CB, cord blood; CMA, chromosomal microarray analysis; chr, chromosome; US, ultrasound; IUFD, intrauterine fetal death; FGR, fetal growth restriction; NT, nuchal translucency; NB, nasal bone.

Table S2. Description of other mosaic chromosomal aberrations^a^ in fetuses with and without ultrasound anomalies

| No. | MA  years | GA  weeks | specimen source | indication(s) | karyotype | CMA |
| --- | --- | --- | --- | --- | --- | --- |
| **cases without US anomalies** | | | | |  |  |
| 36 | 26 | 12^+1^ | CVS | parental thalassemia | 46,XX,del(1)(q11)[6]/46,XY[44] | - |
| 99 | 30 | 19 | CVS/AF | maternal balanced translocation | CVS: 46,XN,del(1)(q11)[7]/46,XN[14] | AF: normal |
| 33 | 36 | 18^+5^ | AF | high risk of NIPT for chr 3; advanced MA | 46,X,?,del(X)(q22.1q22.3)[4]/46,XX[46] | - |
| 24 | 40 | 17 | AF | parental thalassemia; advanced MA; adverse pregnancy history | 46,XX,del(2)(q22q32)[25]/46,XX[25] | - |
| 102 | 35 | 23 | AF/CB | advanced MA | AF: 46,XN,+del(1)(q12)[13]/46,XN[37] | CB: normal |
| 34 | 32 | 20 | AF | high risk of NIPT for chr X | 46,X,psu idic(X)(q22.1)[46]/45,X[4] | - |
| 3 | 32 | 27^+6^ | CB | paternal chromosomal abnormalities | 47,XX,r(Y)(p11q12)[54]/47,XX,dicr(Y;Y)(p11q12;q12p11)[5]/46,XX[41] | - |
| 13 | 31 | 22 | AF/CB | high risk of MSS for T21 | AF:45,X,1qh+[18]/46,X,i(Y)?[12]  CB: 46,X,idic(Y)(q11.23)[34]/45,X[12]/47,X,idic(Y)(q11.23)×2[4] | - |
| 29 | 40 | 23^+4^ | AF/CB | high risk of MSS for T21; advanced MA | AF:46,X,i(X)(q10)[11]/45,X[13]  CB: 45,X[29]/46,X,i(X)(q10)[23] | - |
| 12 | 34 | 19^+1^ | AF | high risk of MSS for T21 | 46,XY,add(2)(pter→q37:?)[1]/46,XY[49] | - |
| 98 | 40 | 23^+6^ | AF/CB | advanced MA | AF: 46,XN,1qh+,?ins(10;1)(q22;q44q21)[15]/ 46,XN,1qh+[89] | CB: normal |
| 42 | 39 | 20^+6^ | AF | advanced MA | 47,XX,+mar[33]/46,XX[22] | - |
| **cases with US anomalies** | | | | |  |  |
| 39 | 28 | 28^+5^ | CB | US: fetal cardiac defect | 46,XY,der(18)t(2;18)(p14;q21.2)[5]/46,XY[45] | - |
| 41 | 35 | 17^+5^ | AF | US: fetal thickened NT; advanced MA | 46,XX,inv(1)(p36.3q42.1)[3]/46,XX[47] | - |
| 19 | 37 | 24 | AF | US: fetal cardiac defect; advanced MA | 47,XX,+psu idic(9)(q21)[27]/46,XX[23] | - |
| 104 | 39 | 20^+6^ | AF/CB | US: fetal thickened NT; advanced MA | AF: 47,XY,+mar[13]/46,XY[37]  CB: 47,XY,+mar[27]/46,XY[23] |  |
| 100 | 28 | 24^+3^ | AF/CB | US: fetal thickened NT | 46,XX[63]/46,XX,der(13;13)(q10;q10),+mar[4] | CB: normal |
| 14 | 30 | 27^+5^ | AF | DCDA  F1: agenesis of corpus callosum;  F2: normal | F1:46,XY,add(10)(q26)[1]/46,XY[19]  F2: normal | - |

Other mosaic chromosomal aberrations^a^: mosaic chromosomal aberrations were listed in this table, not including aneuploidies, bisexual chromosomes and those have been listed in Table S1.

No., number; MA, maternal age; GA, gestational age; CVS, chorionic villus sampling; AF, amniotic fluid; CB, cord blood; CMA, chromosomal microarray analysis; chr, chromosome; US, ultrasound; FGR, fetal growth restriction; NT, nuchal translucency; NB, nasal bone; MSS, maternal serum screening; T, trisomy; DCDA, dichorionic diamniotic; F, fetus.

Table S3. Comparisons of mosaic fraction between cases with and without ultrasound anomalies

|  | US anomalies | | *p* value^a^ |
| --- | --- | --- | --- |
|  | No(n=52) | Yes(n=52) |  |
| **Mosaic Fraction associated with specimen** | mean±SD | mean±SD |  |
| CVS | 0.27±0.25 | 0.56±0.34 | 0.140 |
| AF | 0.34±0.25 | 0.42±0.26 | 0.298 |
| CB | 0.40±0.19 | 0.38±0.23 | 0.747 |
| **Mosaic Fraction associated with overall classification** | mean±SD | mean±SD |  |
| Mosaic aneuploidies | 0.36±0.29 | 0.47±0.29 | 0.190 |
| Mosaic other abnormal karyotypes^b^ | 0.32±0.23 | 0.32±0.30 | 0.988 |
| Mosaic CNVs | 0.42±0.19 | 0.36±0.12 | 0.273 |

Independent-sample T tests were used and all *p* values are two-sided. *p* value^a^: comparisons were performed between cases with and without ultrasound anomalies.

Mosaic other abnormal karyotypes^b^ refer to abnormal karyotypes detected by G-banding karyotyping such as isochromosomes, marker chromosomes, but not including aneuploidies.

US, ultrasound; CVS, chorionic villus sampling; AF, amniotic fluid; CB, cord blood; CNVs, copy number variants.

Table S4. Comparisons of mosaic fractions in aneuploidies and pathogenic CNVs both detected in cases with and without ultrasound anomalies

| chromosomal abnormalities | US anomalies | | *p* vaule^a^ |
| --- | --- | --- | --- |
|  | No(n=52) | Yes(n=52) |  |
| **aneuploidies** | mosaic fractions | mosaic fractions |  |
| 45,X/46,XN | 0.24±0.18(n=9) | 0.41±0.22(n=9) | 0.090 |
| 47,XXX(47,XXY)(47,XYY)/46,XN^b^ | 0.35±0.32(n=7) | 0.44(n=1) | - |
| 45,X/47,XXX^c^ | [4]/[1](n=1) | [34]/[16](n=1) | - |
| 47,XN,+21 | 0.66±0.32(n=3) | 0.68±0.39(n=5) | 0.934 |
| 47,XN,+2 | 0.30(n=1) | 0.51(n=1) | - |
| **pCNVs** |  |  |  |
| 22q11 microduplication syndrome | 0.62±0.21(n=2) | 0.57(n=1) | - |
| **other** |  |  |  |
| XY/XX^‡^ | [26]/[13]  [33]/[17]  [37]/[13]  (n=3) | [14]/[36]  [7]/[21]  [40]/[10]  (n=3) | - |

If the case number in two groups were both more than one, independent-sample T tests were used to compare the data and all *p* values are two-sided.

*p* value^a^: comparisons were performed between cases with and without ultrasound anomalies.

47,XXX(47,XXY)(47,XYY)/46,XN^b^: these karyotypes included 47,XXX/46,XN, 47,XXY/46,XN and 47,XYY/46,XN.

45,X/47,XXX^c^ and XY/XX^c^: mosaic fractions were not calculated into percentages in these mosaic types because confirmation of the true fetal karyotypes was not performed in these cases.

US, ultrasound; pCNVs, pathogenic copy number variants.

Table S5. Chromosomal mosaicism detected in chorionic villus sampling

| No. | MA | GA | indication(s) | karyotype | CMA |
| --- | --- | --- | --- | --- | --- |
| **cases without US anomalies** | | | | | |
| 2 | 22 | 13^+6^ | parental thalassemia | CVS:45,X[30]/46,XY[20]  AF: 45,X[23]/46,XY[28] | - |
| 4 | 32 | 13^+4^ | parental thalassemia | CVS:46,XX[13]/46,XY[26] | - |
| 11 | 26 | 14 | parental thalassemia | CVS:47,XXX[1]/46,XX[49] | - |
| 28 | 34 | 12^+4^ | parental thalassemia | CVS:46,XY[33]/46,XX[17] | - |
| 32 | 35 | 13 | parental thalassemia, advanced MA | CVS:45,X[5]/46,XY[36] | - |
| 36 | 26 | 12^+1^ | parental thalassemia | CVS:46,XX,del(1)(q11)[6]/46,XY[44] | - |
| 48 | 32 | 11^+5^ | parental thalassemia, adverse pregnancy history | - | CVS:Xp22.3q28(2558185-155233731)×1.19,(Y)×1 |
| 51 | 36 | 12 | advanced MA, adverse pregnancy history | - | CVS: 22q11.1q13.33(16888889-51197766) ×2.77 |
| **cases with US anomalies** | | | | | |
| 15 | 29 | 12^+6^ | hydrops fetalis | CVS:45,X[14]/46,XX[26] | - |
| 16* | 30 | 11^+2^ | F1: normal  F2: acardiac defect | CVS: 46,XX[21]/46,XY[7] | - |
| 57 | 28 | 12^+6^ | cystic hygroma | - | CVS: (X) ×1.0-1.2；Ypterq11.223(0-24770883) ×1；Yq11.223q11.23(24770884-28339599) ×2 |
| 60 | 25 | 12 | thickened NT | - | CVS: 18×2.92 |

case 16 was a monochorionic diamniotic twin pregnancies which has only one placenta.

No., number; MA, maternal age; GA, gestational age; CVS, chorionic villus sampling; AF, amniotic fluid; CMA, chromosomal microarray analysis; US, ultrasound; NT, nuchal translucency; F, fetus.
